# Supplementary material for: Meditation and Five Precepts Mediate the Relationship between Attachment and Resilience
Source: Children (Basel). 2022 Mar 7;9(3):371. doi: 10.3390/children9030371 (PMC8947555; doi:10.3390/children9030371)
Supplement: Supplementary file 1 [file children-09-00371-s001.zip › children-1522782 Table S1. PPQ.pdf]

Table S1. Precept Practice Questionnaire (PPQ)

INSTRUCTIONS: Indicate how often each of the statements below is descriptive of you.

Statement	Never	Almost never	Sometimes	Often	Regular
1. I avoid harming living things (including animals and insects).	1	2	3	4	5
2. I avoid taking thinks from people that they have not given me.	1	2	3	4	5
3. I avoid sexual misconduct.	1	2	3	4	5
4. I avoid telling lies.	1	2	3	4	5
5. I avoid alcohol and substance abuse.	1	2	3	4	5
How are you motivated to avoid these behaviors? (Why don’t you do it?) Choose one.					
1. I don’t understand why I don’t do these behaviors	☐				
2. I think they are good for everyone	☐				
3. I want people to think I’m a good person	☐				
